# Integrating multi-omics and reverse network toxicology to identify pollutant risks and potential intervention targets in pulmonary arterial hypertension

**DOI:** 10.3389/fcell.2026.1840760

**Published:** 2026-06-05

**Authors:** Shuying You, Yinhui Sun, Na Li, Hong Xie, Xiangbo Zeng, Xianli Su, Dan liu, Chun Shao, Aiguo Dai

**Affiliations:** 1 Department of Respiratory and Critical Care Medicine, The Second People’s Hospital of Hunan Province (Hunan Provincial Brain Hospital), Changsha, Hunan, China; 2 College of Integrated Traditional Chinese and Western Medicine, Hunan University of Chinese medicine, Changsha, China; 3 Hunan Provincial Key Laboratory of Vascular Biology and Translational Medicine, Changsha, Hunan, China

**Keywords:** environmental pollution risk, idiopathic pulmonary arterial hypertension, multi-omics, reverse network toxicology, SMR analysis

## Abstract

**Background:**

Idiopathic pulmonary arterial hypertension (PAH) is a progressive cardiovascular disorder with high mortality. Although both genetic and environmental factors are implicated in its pathogenesis, the underlying mechanisms remain unclear.

**Methods:**

We integrated transcriptomic data from PAH lung tissue, GWAS summary statistics, and QTL data for DNA methylation, gene expression, and plasma protein. Core dysregulated genes were first identified via differential expression and protein-protein interaction network analysis. Using summary-data-based Mendelian randomization (SMR), we systematically evaluated potential potential genetic associations between methylation, expression, or protein levels of candidate genes and PAH risk, with the HEIDI test to distinguish causality from pleiotropy. Key findings were validated by examining gene expression trends in two independent cohorts. Finally, reverse network toxicology was applied: environmental pollutants targeting identified genes were screened using the CTD, their binding potential assessed via molecular docking, and effects of a candidate pollutant on gene expression and cell proliferation validated *in vitro* in human pulmonary artery smooth muscle cells (HPASMCs).

**Results:**

We identified 254 differentially expressed genes in PAH. Among these, TAGLN2 exhibited a significant positive association with PAH risk at three molecular levels—DNA methylation, gene expression, and plasma protein, suggesting a pathogenic role. Specifically, increased TAGLN2 protein abundance (HR = 9.00, 95% CI: 1.52–53.16) and gene expression levels (HR = 9.00, 95% CI: 1.52–53.16) were associated with higher PAH risk, while its methylation sites (e.g., cg13892570, cg16107628) showed a negative association. Validation in two independent cohorts confirmed that TAGLN2 expression was upregulated in the lung tissue of PAH patients. Reverse toxicology predicted eight environmental pollutants as potential TAGLN2-targeting agents, including PFOS, dibutyl phthalate, bisphenols, and benzo[a]pyrene. Molecular docking indicated that all these compounds could bind stably to the TAGLN2 protein (binding free energy < −5.0 kcal/mol), with PFOS exhibiting the strongest binding affinity (−8.9 kcal/mol). *In vitro* experiments showed that PFOS upregulated TAGLN2 mRNA expression in HPASMCs and promoted cell proliferation in a dose-dependent manner, providing preliminary correlative evidence.

**Conclusion:**

This study prioritizes TAGLN2 as a genetically associated candidate gene for PAH and identifies environmental pollutants that may target TAGLN2. While the *in vitro* data show that PFOS upregulates TAGLN2 expression and promotes HPASMC proliferation, functional perturbation experiments are needed to establish a mechanistic requirement for TAGLN2. These findings provide hypothesis-generating insights into potential gene-environment interactions in PAH.

## Introduction

Idiopathic pulmonary arterial hypertension (PAH) is a chronic and severe progressive disease clinically defined by a sustained elevation of pulmonary artery pressure, often accompanied by right ventricular hypertrophy (Kovacs et al., 2024; Ruopp and Cockrill, 2022). Epidemiological data indicate that the prevalence among adults in the United States is approximately 10.6 cases per million (Ruopp and Cockrill, 2022; Lau et al., 2017). Current therapies targeting the nitric oxide, prostacyclin, and endothelin pathways can slow disease progression and improve outcomes to some extent. However, aside from lung transplantation, there is no cure, and the overall prognosis remains poor (Lau et al., 2017; Pan et al., 2023; Moutchia et al., 2024).

The pathogenesis of PAH is complex, involving interactions among multiple factors such as genetic susceptibility, epigenetic regulation, metabolic abnormalities, and immune-inflammatory responses, which collectively drive pathological pulmonary vascular remodeling (Evans et al., 2021; Olsson et al., 2023; Naeije et al., 2022; Sofianopoulou et al., 2019). Key pathological processes include pulmonary vasoconstriction, aberrant proliferation of human pulmonary artery smooth muscle cells (HPASMCs), and a shift toward an apoptosis-resistant phenotype (Olsson et al., 2023; Naeije et al., 2022). Furthermore, a growing body of evidence suggests that early-life environmental exposures may exacerbate disease progression in genetically predisposed individuals (Sofianopoulou et al., 2019; Long et al., 2025). However, current research has largely focused on single exposures, whereas in reality, individuals are exposed to complex mixtures of chemicals. The potential additive or synergistic effects of these mixtures pose significant challenges for toxicological assessment and etiological clarification (Sofianopoulou et al., 2019; Long et al., 2025). Therefore, identifying key combinations of environmental exposures and their molecular targets is crucial for deepening our understanding of PAH pathogenesis and for guiding the development of evidence-based public health policies.

To address these research gaps, integrative computational strategies have emerged as essential tools for unraveling the etiology of complex diseases (Li et al., 2023; Zhu et al., 2025; Qi et al., 2025; Kan et al., 2017). Summary-data-based Mendelian randomization (SMR) analysis enables a systematic evaluation of how genetic variants influence disease risk by regulating molecular phenotypes such as DNA methylation, gene expression, and protein abundance, thereby identifying potential causal genes (Li et al., 2023). When combined with reverse network toxicology, this approach can further predict environmental exposures that may target these genes or pathways and assess their toxic potential (Zhu et al., 2025; Qi et al., 2025). This integrated “gene-environment” analysis framework provides a powerful tool for exploring the interplay between environmental exposures and genetic susceptibility in the pathogenesis of PAH (Li et al., 2023; Zhu et al., 2025; Qi et al., 2025).

In this study, we therefore propose to systematically investigate these mechanisms using an integrative computational approach. First, we will integrate multi-omics data, including DNA methylation, gene expression, and protein abundance, to construct instrumental variables. SMR analysis will be employed to identify genes potentially associated with PAH risk via genetic regulation, and key findings will be validated through *in vitro* experiments. Subsequently, we will apply reverse network toxicology to these identified genes for molecular target prediction and molecular docking analysis, aiming to pinpoint key environmental pollutants that may contribute to PAH development. This work is intended to provide a theoretical basis for the formulation of personalized strategies for the prevention and treatment of PAH.

## Materials and methods

### Bulk sequencing data analysis

Two PAH-related microarray datasets, GSE117261 and GSE24988, were obtained from the GEO database. GSE117261 includes samples from 58 PAH patients and 25 controls, while GSE24988 comprises samples from 94 PAH patients and 22 controls. Differential expression analysis between the control and idiopathic PAH groups was performed using the “limma” package (version 3.50.0). Specifically, the lmFit function was used to construct linear models fitting the gene expression data, followed by empirical Bayes correction with the eBayes function to enhance the robustness of statistical inference. Differentially expressed genes (DEGs) were identified based on the criteria of |log_2_ fold change| > 0.65 and an adjusted p-value <0.05.

### Summary statistics for eQTL, mQTL, and pQTL

The QTL data utilized in this study were derived from the following high-quality cohorts: Gene expression QTL (eQTL) data were obtained from the eQTLGen consortium, integrating 37 studies with a total of 31,684 samples and encompassing 10,317 SNPs associated with gene expression regulation (Võsa et al., 2021). DNA methylation QTL (mQTL) data were sourced from the study by McRae et al., which included 1,980 samples from two European cohorts (McRae et al., 2018). Plasma protein QTL (pQTL) data were obtained from an Icelandic cohort comprising 35,559 participants and 4,907 proteins (Sun et al., 2023).

### PAH outcome dataset

In this study, genome-wide association study (GWAS) data for PAH were obtained from the FinnGen project (https://www.finngen.fi/en). FinnGen is a large-scale genomics initiative that has analyzed over 500,000 Finnish biobank samples. By integrating genetic variant data with health records, the project aims to elucidate disease mechanisms and individual susceptibility. This collaborative effort involves Finnish research institutions, biobanks, and international industry partners (Kurki et al., 2023). The FinnGen dataset used here includes 301 PAH cases and 345,634 control individuals.

### Functional enrichment analysis

Gene Ontology (GO; http://geneontology.org) categorizes gene functions into three domains: Cellular Component (CC), Molecular Function (MF), and Biological Process (BP). The Kyoto Encyclopedia of Genes and Genomes (KEGG; https://www.kegg.jp) systematically links genomic information to higher-order functional pathways. GO and KEGG enrichment analyses were performed using the R package clusterProfiler.

### Protein-protein interaction (PPI) network construction

A PPI network was constructed using the STRING database with a confidence threshold of ≥0.4. After filtering to remove disconnected nodes, the network data were imported into Cytoscape (version 3.9.0) for visualization. Topological parameters, including degree centrality, closeness centrality, and betweenness centrality, were calculated using the CentiScaPe 2.0 plugin. Nodes were ranked by their closeness centrality values, where a higher value indicates a more central and potentially critical role within the network.

### Summary-data-based mendelian randomization (SMR)

In this study, we employed SMR to prioritize genes potentially associated with genetic susceptibility to PAH. SMR integrates GWAS summary statistics for an exposure (e.g., QTL) and an outcome (PAH) from large, independent samples, using significant cis-quantitative trait loci (cis-QTLs) as instrumental variables. Compared to traditional MR methods, SMR often provides greater statistical power (Li et al., 2023). To distinguish potential causal associations from those driven by pleiotropy, we conducted the HEIDI test. A p-value less than 0.05 in the HEIDI test suggests that the association may be confounded by linkage disequilibrium (LD) and does not meet the assumptions for inferring a potential genetic association; such associations were excluded from subsequent analyses (Zhu et al., 2016). LD reference data were obtained from the 1000 Genomes Project. All SMR and HEIDI analyses were performed using the SMR software (version 1.3.1, https://yanglab.westlake.edu.cn) developed by Yang’s lab.

### Environmental pollutant prediction

To identify environmental pollutants relevant to PAH, the Comparative Toxicogenomics Database (CTD) was queried using the previously identified causal genes as bait. To ensure biological relevance and specificity of the screening, the following inclusion and exclusion criteria were applied: Only compounds with a clearly documented unidirectional regulatory effect on the target gene in the CTD, and where this effect direction was consistent with the gene’s dysregulation pattern observed in PAH, were retained. Compounds exhibiting bidirectional regulation (both activation and inhibition) or those influencing gene expression solely through indirect pathways were excluded.

### Toxicological property prediction

To systematically evaluate the toxicological profiles of the candidate compounds, two specialized computational platforms were employed for toxicity prediction: ADMETlab 3.0 (Fu et al., 2024; Banerjee et al., 2024) was used to predict absorption, distribution, metabolism, and excretion (ADME) properties, and the ProTox-III platform was used for multi-endpoint toxicity assessment. Only compounds predicted to be absorbable and with a potential for accumulation were advanced to subsequent analyses.

### Molecular docking

To investigate potential interactions between the screened environmental pollutants and the target protein, molecular docking simulations were performed. The two-dimensional structures of the ligand molecules (environmental pollutants) were obtained from the PubChem database, and the three-dimensional structure of the key target protein was retrieved from the AlphaFold Protein Structure Database. Structural optimization and docking simulations for both ligands and the receptor were conducted using AutoDockTools (version 1.5.7) to predict binding modes and calculate the binding free energy (ΔG), thereby assessing the stability and potential functional impact of the interaction. A binding free energy below 0 kcal/mol indicates a spontaneous binding process, while a value below −5.0 kcal/mol suggests the formation of a stable complex.

### Cell culture and treatment

Human pulmonary artery smooth muscle cells (HPASMCs) were obtained from ScienCell Research Laboratories. Cells from passages 4 to 9 were used in this study. Cells were cultured in complete smooth muscle cell medium and maintained at 37 °C in a humidified incubator with 5% CO_2_. Cells were expanded for 3 to 8 passages for use in subsequent experiments. According to the experimental design and based on previous literature (Yue et al., 2026; Xu et al., 2025), HPASMCs were treated with 0, 50, or 100 µM of PFOS for 24 h. The concentrations were selected based on prior studies in vascular smooth muscle cells (Yue et al., 2026; Xu et al., 2025), where 100 µM represented the upper limit without significant cytotoxicity. All experiments were performed with three independent biological replicates, each with technical triplicates.

### Quantitative real-time PCR (qRT-PCR) analysis

Total RNA was isolated from cells using the EZ-press RNA Purification Kit (EZBioscience, China). RNA purity was verified using a NanoDrop 2000 spectrophotometer, with an acceptable A260/A280 ratio >1.8. High-quality RNA was reverse transcribed into cDNA using the PrimeScript™ RT Reagent Kit. Quantitative PCR was subsequently performed on a QuantStudio 5 system using the Premix Ex Taq™ Kit under the following cycling conditions: initial denaturation at 95 °C for 30 s, followed by 40 cycles of denaturation at 95 °C for 5 s and annealing/extension at 60 °C for 34 s. All reactions were performed in triplicate.

### EdU proliferation assay

Cell proliferation was assessed using the Cell-Light™ EdU Apollo® 488 *In Vitro* Imaging Kit (Beyotime, China). HPASMCs were seeded in 24-well plates at a density of 3 × 10^4^ cells per well and cultured until they reached 70%–80% confluence. Cells were then incubated with 20 µM EdU working solution (Servicebio, China) for 2 h at 37 °C in a 5% CO_2_ atmosphere. Subsequently, cells were fixed with 4% paraformaldehyde for 15 min and permeabilized with 0.5% Triton X-100 for 20 min. Proliferating cells with incorporated EdU were fluorescently labeled using the Click-iT™ EdU Alexa Fluor™ 594 Imaging Kit (Thermo Fisher Scientific, United States of America) according to the manufacturer’s instructions. Fluorescent images were captured using a Nikon Eclipse Ts2R FL inverted fluorescence microscope with a ×20 objective.

### Statistical analysis

All statistical analyses were performed using R software (version 4.2.2). Differences in gene expression were evaluated using the non-parametric Wilcoxon signed-rank test and the parametric paired Student’s t-test. For PFOS dose-response experiments (qPCR and EdU), one-way analysis of variance (ANOVA) followed by Tukey’s post-hoc test was used to compare multiple groups. Statistical significance was defined as a two-sided p-value <0.05. All *in vitro* experiments were conducted with a minimum of three independent replicates, and each replicate included technical triplicates.

## Results

### Identification and enrichment analysis of dysregulated genes in PAH

**FIGURE 1:**
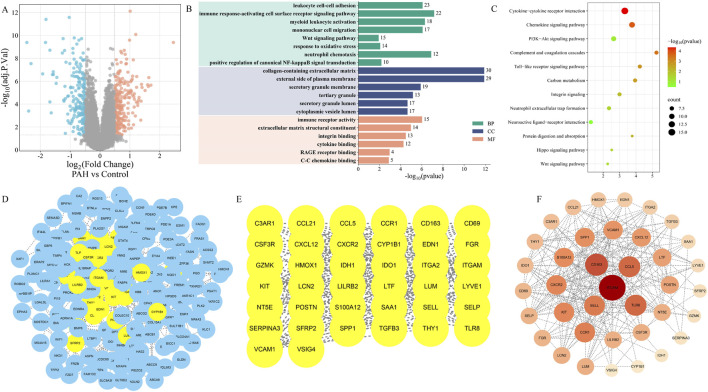
Identification and enrichment analysis of differentially expressed genes (DEGs) in pulmonary arterial hypertension (PAH). **(A)** Volcano plot of DEGs between PAH patients (n = 58) and controls (n = 25) in the GSE117261 cohort. Differential expression was analyzed using the limma package (version 3.50.0) with empirical Bayes moderation; significance criteria: |log_2_FC| > 0.65 and Benjamini–Hochberg adjusted P < 0.05. Red and blue dots represent significantly upregulated and downregulated genes, respectively. **(B)** Gene Ontology (GO) enrichment analysis of the 254 DEGs, showing top enriched biological processes, cellular components, and molecular functions. **(C)** KEGG pathway enrichment analysis of the DEGs, highlighting pathways related to inflammation, oxidative stress, and cell proliferation. **(D)** Protein-protein interaction (PPI) network of the DEGs constructed using STRING (confidence ≥0.4) and visualized in Cytoscape. Node size and color intensity are proportional to degree centrality. **(E)** Topological ranking of hub genes based on degree centrality. **(F)** Degree scores of the five hub genes with the highest connectivity (ITGAM, CD163, CCL5, SELL, TLR8).

First, differential expression analysis of the GSE117261 microarray cohort identified 254 differentially expressed genes (DEGs) in PAH patient samples compared to controls, comprising 137 upregulated and 117 downregulated genes (Figure 1A). These genes were subsequently identified as potential regulators involved in PAH pathogenesis. Gene Ontology (GO) enrichment analysis revealed that these genes were primarily involved in the regulation of inflammatory pathways, the Wnt signaling pathway, and responses to oxidative stress (Figure 1B). Further KEGG pathway analysis indicated significant associations with various pathways related to inflammation, oxidative stress, and cell proliferation and migration (Figure 1C).

### Protein-protein interaction (PPI) network construction of PAH dysregulated genes

The 254 PAH-related DEGs were imported into the STRING database to construct a protein-protein interaction (PPI) network, using a confidence threshold of ≥0.4. After removing isolated nodes, a network comprising 247 target proteins was obtained. The network was visualized using Cytoscape (version 3.10.3), with node size and color intensity mapped to their degree of connectivity; higher connectivity is represented by larger node diameters and darker colors. Topological analysis further identified the five nodes with the highest degree of connectivity as core targets: ITGAM, CD163, CCL5, SELL, and TLR8 (Figures 1D–F). This network illustrates the interactions among these key targets and provides clues for further exploring the molecular mechanisms underlying PAH pathogenesis.

### Association of blood protein abundance of PAH dysregulated genes with PAH genetic susceptibility

**FIGURE 2:**
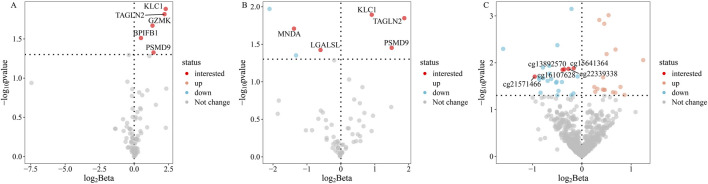
Summary-data-based Mendelian randomization (SMR) analysis identifies genes associated with PAH risk. **(A)** Forest plot of SMR estimates for the association between plasma protein abundance (pQTL) of five genes (TAGLN2, GZMK, PSMD9, KLC1, BPIFB1) and PAH risk. Hazard ratios (HR) and 95% confidence intervals are shown. **(B)** Forest plot of SMR estimates for the association between blood gene expression levels (eQTL) of five genes (MNDA, TAGLN2, CD14, PSMD9, KLC1) and PAH risk. **(C)** Summary of SMR results for DNA methylation (mQTL), depicting the 39 CpG sites within 17 genes that are significantly associated with PAH risk (23 risk-increasing, 16 risk-decreasing). All associations passed the HEIDI test (P > 0.05).

After screening, 91 of the PAH dysregulated genes had valid instrumental variables in the pQTL data. Following SMR analysis (p < 0.05) and subsequent validation using the HEIDI test (p > 0.05) to distinguish causality from pleiotropy, the blood protein abundance of five PAH dysregulated genes (TAGLN2, GZMK, PSMD9, KLC1, and BPIFB1) was found to be significantly associated with genetic susceptibility to PAH (Figure 2A; Table 1; Supplementary Table S3). Specifically, increased abundance of the proteins encoded by all five genes was associated with an elevated risk of PAH: TAGLN2 (HR = 9.00, 95% CI: 1.52–53.16, p = 0.015), GZMK (HR = 3.78, 95% CI: 1.22–11.72, p = 0.021), PSMD9 (HR = 4.07, 95% CI: 1.02–16.27, p = 0.047), KLC1 (HR = 9.85, 95% CI: 1.62–60.06, p = 0.013), and BPIFB1 (HR = 1.64, 95% CI: 1.05–2.56, p = 0.021).

**TABLE 1:** The association of protein abundance of PAH dysregulated genes with genetic susceptibility to PAH.

Gene	HR	LCI	UCI	P for SMR	P for HEIDI
TAGLN2	8.81	1.52	51.17	0.015	0.559
GZMK	3.78	1.22	11.72	0.021	0.253
PSMD9	4.07	1.02	16.27	0.047	0.301
KLC1	9.85	1.62	60.06	0.013	0.512
BPIFB1	1.64	1.05	2.56	0.031	0.152

### Association of blood gene expression levels of PAH dysregulated genes with PAH genetic susceptibility

Following screening, 56 of the PAH dysregulated genes had valid instruments in the eQTL data. SMR analysis (p < 0.05) combined with HEIDI test validation (p > 0.05) revealed that the blood gene expression levels of five PAH dysregulated genes (MNDA, TAGLN2, CD14, PSMD9, and KLC1) were significantly associated with PAH genetic susceptibility (Figure 2B; Table 2; Supplementary Table S4). Specifically, increased expression of three genes was associated with higher PAH risk: TAGLN2 (HR = 9.00, 95% CI: 1.52–53.16, p = 0.015), PSMD9 (HR = 4.07, 95% CI: 1.02–16.27, p = 0.047), and KLC1 (HR = 9.85, 95% CI: 1.62–60.06, p = 0.013), while increased expression of two genes (MNDA and CD14) was associated with decreased risk.

**TABLE 2:** The association of expression levels of PAH dysregulated genes with genetic susceptibility to PAH.

Gene	HR	LCI	UCI	P for SMR	P for HEIDI
MNDA	0.25	0.08	0.80	0.020	0.670
TAGLN2	6.49	1.45	28.92	0.014	0.969
CD14	0.27	0.07	0.97	0.045	0.305
PSMD9	4.47	1.11	18.02	0.035	0.782
KLC1	2.49	1.21	5.09	0.013	0.782

### Association of blood methylation sites of PAH dysregulated genes with PAH genetic susceptibility

After screening, valid mQTL data were available for a subset of the 786 methylation sites associated with 173 PAH dysregulated genes. SMR analysis (p < 0.05) followed by HEIDI test validation (p > 0.05) identified 39 CpG sites within 17 genes that were significantly associated with PAH genetic susceptibility. Among these, 23 CpG sites were associated with an increased risk of PAH, and 16 CpG sites were associated with a decreased risk (Figure 2C; Table 3; Supplementary Table S5).

**TABLE 3:** The association of methylation sites of PAH dysregulated genes with genetic susceptibility to PAH.

Methylation site	Gene	HR	LCI	UCI	P for SMR	P for HEIDI
cg11227872	CSF3R	0.52	0.30	0.89	0.017	0.808
cg22339338	TAGLN2	0.76	0.61	0.95	0.014	0.552
cg15641364	TAGLN2	0.85	0.74	0.97	0.013	0.633
cg13892570	TAGLN2	0.70	0.53	0.93	0.014	0.771
cg16107628	TAGLN2	0.68	0.49	0.92	0.014	0.740
cg21571466	TAGLN2	0.38	0.17	0.86	0.020	0.722
cg24351977	LTBP1	1.98	1.23	3.20	0.005	0.241
cg03906115	LTBP1	3.47	1.37	8.82	0.009	0.151
cg13048967	CXCR1	1.72	1.25	2.37	0.001	0.939
cg06683602	CXCR1	0.81	0.72	0.92	0.001	0.548
cg26010218	FRAS1	0.61	0.37	1.00	0.050	0.311
cg17082661	FRAS1	0.43	0.19	0.98	0.044	0.610
cg07442568	FRAS1	2.19	1.07	4.50	0.033	0.863
cg11000292	ABCG2	0.50	0.27	0.91	0.024	0.276
cg24352530	ABCG2	0.54	0.31	0.91	0.022	0.272
cg01817885	PDE4D	0.84	0.71	1.00	0.045	0.219
cg04580929	PDE4D	0.81	0.65	1.00	0.050	0.874
cg12192566	CD14	1.57	1.03	2.39	0.038	0.158
cg14958663	CD14	1.37	1.02	1.84	0.036	0.138
cg02343503	CD14	2.37	1.01	5.61	0.049	0.636
cg12143439	CD14	1.59	1.03	2.48	0.038	0.053
cg26671749	SLC36A1	0.61	0.43	0.85	0.004	0.335
cg12546582	PDE7B	0.45	0.24	0.84	0.013	0.355
cg21817179	SOSTDC1	0.59	0.37	0.94	0.027	0.794
cg25533774	SOSTDC1	0.67	0.47	0.95	0.026	0.547
cg10378667	PSMD9	0.60	0.38	0.94	0.026	0.523
cg01797106	GLT1D1	0.42	0.20	0.88	0.021	0.310
cg07925824	GLT1D1	1.65	1.15	2.37	0.007	0.727
cg23090046	KLC1	0.52	0.31	0.86	0.012	0.848
cg17925084	KLC1	1.42	1.15	1.76	0.001	0.145
cg11828104	KLC1	1.58	1.19	2.09	0.001	0.174
cg12112151	KLC1	0.20	0.07	0.62	0.005	0.254
cg02907524	AGBL1	1.31	1.01	1.70	0.042	0.752
cg26556336	AGBL1	0.92	0.85	0.99	0.020	0.483
cg09159068	CFD	1.97	1.02	3.81	0.044	0.236
cg23514110	CFD	1.88	1.02	3.45	0.043	0.383
cg02447462	CFD	1.29	1.02	1.63	0.033	0.168
cg22152605	FPR1	0.62	0.40	0.98	0.040	0.588

Notably, the three genes (TAGLN2, PSMD9, and KLC1) whose increased gene and protein expression levels were associated with higher PAH risk also showed significant associations between their CpG sites and PAH susceptibility. Specifically, five CpG sites related to TAGLN2 (cg22339338, cg15641364, cg13892570, cg16107628, and cg21571466) and one CpG site related to PSMD9 (cg10378667) were associated with decreased PAH risk. For KLC1, among its four associated CpG sites, cg23090046 and cg12112151 were linked to decreased risk, whereas cg17925084 and cg11828104 were associated with increased risk. Based on this multi-dimensional evidence from mQTL, pQTL, and eQTL data, TAGLN2, PSMD9, and KLC1 were identified as key targets significantly associated with PAH risk and were prioritized for subsequent in-depth analysis.

### Validation of key dysregulated gene expression in PAH

**FIGURE 3:**
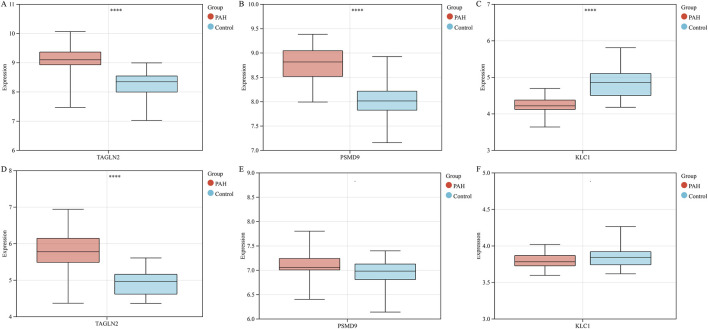
Validation of TAGLN2, PSMD9, and KLC1 expression in independent PAH cohorts. **(A–C)** Expression levels of TAGLN2, PSMD9, and KLC1 in the GSE117261 cohort (58 PAH patients, 25 controls). **(D–F)** Expression levels of the same genes in the GSE24988 cohort (94 PAH patients, 22 controls). Data are presented as mean ± SEM. *P < 0.05, **P < 0.01, ***P < 0.001; ns, not significant (Wilcoxon test or t-test).

To validate whether the expression trends of TAGLN2, PSMD9, and KLC1 in PAH patients were consistent with the SMR analysis results, their expression levels were evaluated in two independent cohorts, GSE117261 and GSE24988. In the GSE117261 cohort, the expression trends for TAGLN2 and PSMD9 were consistent with the SMR findings, showing significant upregulation in PAH patients compared to controls. However, KLC1 exhibited an opposite expression trend (Figures 3A–C). In the validation cohort GSE24988, only TAGLN2 showed significant upregulation in PAH patients, aligning with the SMR results, while PSMD9 and KLC1 did not display significant differential expression (Figures 3D–F). Based on these validation results, TAGLN2 was selected as the primary focus for subsequent in-depth analysis.

#### Initial screening of environmental compounds and molecular docking with TAGLN2

**FIGURE 4:**
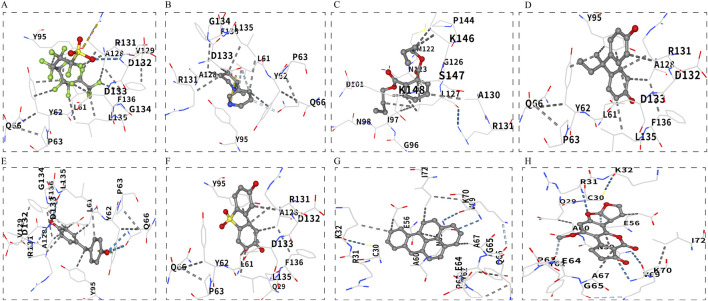
Molecular docking of environmental pollutants with the TAGLN2 protein. **(A–H)** Predicted binding conformations of eight environmental compounds with TAGLN2: **(A)** perfluorooctane sulfonate (PFOS, ΔG = −8.9 kcal/mol), **(B)** nicotine (−5.96 kcal/mol), **(C)** dibutyl phthalate (−5.9 kcal/mol), **(D)** bisphenol B (−6.2 kcal/mol), **(E)** bisphenol S (−6.0 kcal/mol), **(F)** bisphenol F (−6.3 kcal/mol), **(G)** benzo[a]pyrene (−8.1 kcal/mol), **(H)** aflatoxin B1 (−8.3 kcal/mol). TAGLN2 is shown in cartoon representation; key interacting residues are highlighted as sticks. Binding free energies (ΔG) are indicated.

Based on records from the CTD, 137 known compounds capable of modulating TAGLN2 expression were initially identified. These primarily included persistent organic pollutants, endocrine disruptors, pesticides and their metabolites, heavy metals, and air/combustion-related pollutants. Applying the pre-defined screening criteria (unidirectional regulatory effect consistent with the trend from SMR analysis) and incorporating toxicity prediction analysis, eight environmental pollutants were ultimately selected for further study. These compounds, predicted to be inhalable, prone to accumulation in the body, and exhibiting cytotoxic effects, were: perfluorooctane sulfonate (PFOS), nicotine, dibutyl phthalate, bisphenol B, bisphenol S, bisphenol F, benzo[a]pyrene, and aflatoxin B1. Subsequently, molecular docking was performed to assess the binding affinity of these compounds with TAGLN2. The results indicated that all eight compounds could bind stably to the target protein, with binding free energies below −5 kcal/mol: PFOS (−8.9 kcal/mol; Figure 4A), nicotine (−5.96 kcal/mol; Figure 4B), dibutyl phthalate (−5.9 kcal/mol; Figure 4C), bisphenol B (−6.2 kcal/mol; Figure 4D), bisphenol S (−6.0 kcal/mol; Figure 4E), bisphenol F (−6.3 kcal/mol; Figure 4F), benzo[a]pyrene (−8.1 kcal/mol; Figure 4G), and aflatoxin B1 (−8.3 kcal/mol; Figure 4H).

### Effect of PFOS on pulmonary artery smooth muscle cells

**FIGURE 5:**
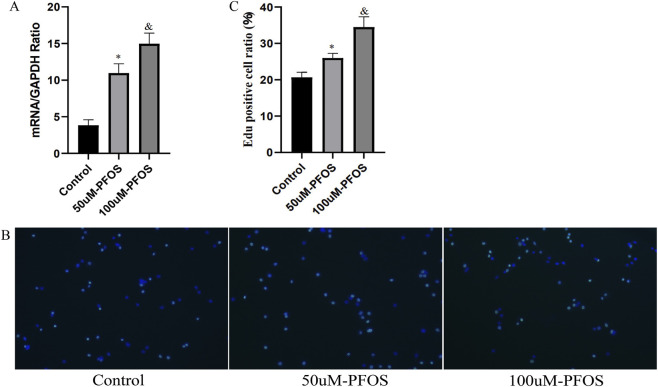
PFOS upregulates TAGLN2 expression and promotes proliferation of human pulmonary artery smooth muscle cells (HPASMCs). **(A)** qRT-PCR analysis of TAGLN2 mRNA levels in HPASMCs treated with 0, 50, or 100 μM PFOS for 24 h. Expression was normalized to GAPDH and is shown as mean ± SD (n = 3 independent experiments, each with technical triplicates). Statistical analysis: one-way ANOVA with Tukey’s post-hoc test. **(B)** EdU proliferation assay. Representative fluorescence images (left) show EdU-positive cells (red) and Hoechst-stained nuclei (blue). Scale bar, 50 μm. **(C)** Quantification of EdU-positive cell ratio (right) is presented as mean ± SD (n = 3 independent experiments). Statistical analysis as in (A/C). *P < 0.05 vs control; #P < 0.05 vs 50 μM PFOS.

To test the predictions from reverse network toxicology and molecular docking, PFOS, which exhibited the most stable binding *in silico*, was selected for *in vitro* experiments. qPCR results showed that PFOS treatment (50 and 100 µM for 24 h) was associated with increased TAGLN2 mRNA levels in HPASMCs (Figure 5A). EdU proliferation assays indicated that PFOS exposure was associated with increased HPASMC proliferation in a dose-dependent manner (Figures 5B,C). These findings are correlative: they demonstrate an association between PFOS treatment and both TAGLN2 upregulation and increased proliferation, but they do not prove that PFOS directly binds to TAGLN2 or that TAGLN2 is mechanistically required for the proliferative effect. Therefore, these results should be considered preliminary and hypothesis-generating. Functional perturbation experiments (e.g., TAGLN2 knockdown or overexpression) are required to test whether TAGLN2 plays a causal role in PFOS-induced proliferation.

## Discussion

PAH is a progressive disease that severely impairs patients’ quality of life. Characterized by persistent elevation of pulmonary artery pressure and right heart failure, it imposes a substantial burden on healthcare systems worldwide (Kovacs et al., 2024; Ruopp and Cockrill, 2022; Lau et al., 2017). Current research indicates that the pathogenesis of PAH is complex, involving interactions among multiple factors including genetic susceptibility, epigenetic regulation, and environmental exposures, which collectively drive pathological pulmonary vascular remodeling (Olsson et al., 2023; Naeije et al., 2022; Sofianopoulou et al., 2019). In real-world scenarios, environmental factors often exist as complex mixtures of multiple chemicals. The potential additive or synergistic effects of these mixtures pose significant challenges for toxicological assessment and etiological clarification (Sofianopoulou et al., 2019; Long et al., 2025). Furthermore, although it is known that genetic and environmental factors jointly contribute to PAH development, the precise interactive mechanisms within specific biological pathways—particularly how they might “program” long-term disease susceptibility during critical early developmental windows—remain incompletely understood (Sofianopoulou et al., 2019; Long et al., 2025).

To address this gap, the present study integrated multi-omics data, Mendelian randomization, and reverse network toxicology to identify and validate the pivotal role of TAGLN2 in PAH. TAGLN2 (Transgelin-2) is an actin-binding protein localized to human chromosome 1q21-q25, first characterized by Stanier et al., in 1998. By binding to actin filaments, this protein participates in regulating dynamic cytoskeletal reorganization and plays a critical role in the contraction and migration of vascular smooth muscle cells (Yan et al., 2025; Yin et al., 2019; Lees-Miller et al., 1987). TAGLN2 is highly expressed not only in smooth muscle and epithelial cells but also in non-smooth muscle cells such as endothelial cells, bone marrow cells, and pancreatic cells. It is widely distributed across various tissues including the lung, ovary, bladder, colon, spleen, and pancreas (Yan et al., 2025; Yin et al., 2019; Lees-Miller et al., 1987). Although previous studies have reported aberrant expression of TAGLN2 in various cardiovascular and pulmonary diseases, including its upregulation in lung tissue from PAH patients where it is considered a potential therapeutic target, the precise mechanisms by which it contributes to PAH pathogenesis warrant further investigation (Zhang et al., 2014; Zhou et al., 2022; Huang et al., 2018; Prattichizzo et al., 2024). The SMR analysis in our study revealed that TAGLN2 was significantly associated with PAH risk at the levels of DNA methylation, gene expression, and plasma protein, suggesting its role as a multi-faceted regulatory hub in PAH. Mechanistic insights indicated that increased TAGLN2 gene and protein abundance correlated with elevated PAH risk, implying a pathogenic role for TAGLN2, whereas several TAGLN2-associated CpG sites were linked to decreased risk. However, whether these methylation sites directly downregulate TAGLN2 expression remains to be established, as we lack paired methylation-expression data in lung tissue.

Furthermore, by querying the CTD, we screened and identified eight environmental pollutants capable of modulating TAGLN2 expression, including PFOS, dibutyl phthalate, bisphenols (bisphenol B, S, and F), benzo[a]pyrene, aflatoxin B1, and nicotine. Although direct epidemiological evidence linking these specific compounds to PAH is currently limited, existing research indicates that these pollutants can induce aberrant proliferation and phenotypic switching of vascular smooth muscle cells in various models of cardiovascular disease. The underlying mechanisms are thought to involve oxidative stress, inflammatory responses, and cytoskeletal remodeling (Sofianopoulou et al., 2019; Long et al., 2025; Prattichizzo et al., 2024; Zago et al., 2022; Münzel et al., 2025; Combes and Franchineau, 2019). Notably, Yue et al. recently reported that PFOS promotes the transition of vascular smooth muscle cells toward a synthetic phenotype via activation of the ERK/tPA signaling pathway, thereby exacerbating atherosclerosis and intimal hyperplasia (Yue et al., 2026; Xu et al., 2025).

Nevertheless, its regulatory effect on TAGLN2 in the context of PAH had not been previously reported. Our study is the first to propose TAGLN2 as a potential molecular target for these environmental pollutants within the pulmonary vasculature. Molecular docking simulations confirmed that all eight compounds could bind stably to the TAGLN2 protein (binding free energy < −5.0 kcal/mol), with PFOS exhibiting the most robust binding affinity (−8.9 kcal/mol). This suggests that PFOS may directly interact with TAGLN2, potentially disrupting the dynamic homeostasis and functional integrity of the smooth muscle cell cytoskeleton, thereby contributing to PAH pathogenesis. Finally, *in vitro* experiments demonstrated that PFOS significantly upregulated TAGLN2 expression and promoted proliferation in pulmonary artery smooth muscle cells. These findings provide crucial clues for future investigations into the mechanistic “environmental exposure-TAGLN2-pulmonary vascular remodeling” axis.

A major strength of this study lies in its multi-faceted integrative strategy, which substantially strengthens the evidence for a potential genetic association between TAGLN2 and PAH risk. First, by converging multi-omics data (DNA methylation, gene expression, plasma protein), *in vitro* functional experiments, and validation in independent microarray cohorts, we established multiple lines of evidence that collectively support the reliability of the associations derived from SMR analysis. The consistent association of TAGLN2 with PAH risk across three molecular levels, its validated expression in two independent cohorts, and the correlative upregulation by PFOS *in vitro* provide a foundation for prioritizing TAGLN2 as a candidate gene for further mechanistic studies. The SMR methodology itself effectively mitigates confounding and helps rule out reverse causation, while the HEIDI test further eliminates false positives potentially arising from linkage disequilibrium. The incorporation of reverse network toxicology and molecular docking techniques provides clues for understanding how mixed environmental pollutants might influence pulmonary vascular remodeling by targeting TAGLN2. Furthermore, this approach opens new avenues for identifying early biomarkers of PAH and formulating targeted environmental intervention strategies.

From a clinical translation perspective, this study suggests that TAGLN2 has potential as an early diagnostic biomarker and an intervention target for PAH. Current diagnosis of PAH largely relies on right heart catheterisation, which often misses the early window. By contrast, TAGLN2 protein and gene expression levels in blood are significantly associated with PAH risk, indicating that liquid biopsy could help identify high-risk individuals or enable early warning. In addition, differences in DNA methylation (such as CpG sites like cg13892570) provide new targets for epigenetic-based early screening, linking molecular findings to the needs of early clinical diagnosis. At the level of preventive medicine, the eight environmental pollutants identified in this study, particularly PFOS, offer clear targets for PAH risk intervention. Individual exposure monitoring for these pollutants (e.g., PFOS, phthalates and bisphenols) could be integrated into health management strategies for high-risk populations, such as those with a family history or carrying genetic risk variants. Public health policies can accordingly be developed to provide guidance on reducing exposure–for example, advising pregnant women and children to avoid certain plastic products or reduce inhalation of polluted air–thereby lowering PAH risk at the source.

These findings also highlight the translational value of gene–environment interactions (G × E). Multi-omic SMR analysis suggests that the genetic regulation (methylation and expression) of TAGLN2 may mediate the effect of environmental exposures on disease. Future research could proceed along two directions. First, prospective cohort studies could be conducted to validate the quantitative relationships between concentrations of pollutants such as PFOS, TAGLN2 expression and PAH risk, thereby clarifying the causal chain of ‘exposure–biomarker–disease’. Second, using pulmonary artery smooth muscle cells derived from induced pluripotent stem cells (iPSCs) carrying specific TAGLN2 genetic variants, *in vitro* G × E models could be established to assess how different pollutants drive abnormal cell proliferation and vascular remodelling under distinct genetic backgrounds. These efforts would provide a scientific basis for precision prevention of PAH–implementing personalised environmental interventions for individuals with specific genetic backgrounds.

It is worth noting that, given the exploratory hypothesis-generating aim of this study, we did not apply formal multiple testing correction (e.g., FDR or Bonferroni) to the SMR results. Overly stringent correction at this screening stage would increase the risk of false negatives and could discard potentially relevant targets. Instead, we employed a multi-evidence convergence strategy to control false positives: (i) the HEIDI test (P > 0.05) excluded associations likely due to linkage disequilibrium or pleiotropy; (ii) priority was given to genes showing consistent associations across at least two molecular levels (mQTL, eQTL, and/or pQTL); (iii) key findings were validated in two independent lung tissue expression cohorts (GSE117261 and GSE24988); and (iv) environmental pollutant predictions were cross-checked for directional consistency. This approach prioritizes biologically plausible signals while maintaining sensitivity for discovery.

Although this study is innovative in integrating multi-omics with environmental exposure prediction, several limitations should be acknowledged. First, regarding causal inference, it is important to clarify that while SMR combined with the HEIDI test can prioritize gene regulatory signals (e.g., at the expression, methylation, or protein level) associated with disease risk, it cannot establish true causality. Residual pleiotropy or linkage disequilibrium may still bias the results, and this method cannot replace functional validation. Therefore, the conclusions concerning TAGLN2 should be regarded as suggestive genetic association evidence rather than definitive causal evidence. Similarly, the *in vitro* PFOS experiment only demonstrates a correlation between TAGLN2 mRNA upregulation and increased cell proliferation, but does not prove that TAGLN2 plays a mechanistically essential role in the proliferative effect; other pathways or genetic associations may still exist. Future experiments involving TAGLN2 knockout or overexpression in pulmonary arterial smooth muscle cells are needed to test whether it is required for pollutant-induced proliferation.

Second, the eQTL, mQTL, and pQTL resources used in this study are derived from blood or plasma cohorts, whereas the differential expression and validation data come from lung tissue of PAH patients. For a disease like PAH, where the primary pathology is located in the pulmonary vasculature, blood-based QTLs may not fully capture the regulatory architecture in diseased lung tissue—genetic variants affecting gene expression or methylation in blood may not have equivalent effects in pulmonary arterial smooth muscle cells or endothelial cells. Nevertheless, it should be acknowledged that after exposure to environmental pollutants (e.g., PFOS, bisphenols, phthalates), these agents are distributed systemically via the blood, and blood molecular profiles may capture systemic responses relevant to vascular pathology. Therefore, despite the limitations of cross-tissue inference, it remains feasible to use blood/plasma QTLs as an initial approach to link environmental exposures to candidate genes. However, the SMR findings of this study should be interpreted as cross-trait genetic associations rather than direct evidence of pulmonary vascular regulation. Future studies should employ lung-tissue or vascular-specific QTL resources to validate these findings in relevant tissue contexts.

Third, although multiple CpG sites (e.g., cg13892570, cg16107628) were found to be significantly associated with PAH risk, this study did not directly demonstrate that these methylation sites regulate TAGLN2 mRNA or protein levels. Because paired methylation-expression quantitative data from PAH lung tissue are lacking, the proposed direction of regulation (e.g., methylation decreasing expression) remains speculative. Thus, the methylation results should be regarded as statistical association signals that provide prioritized CpG sites for subsequent functional studies, rather than established regulatory mechanisms.

Fourth, the environmental pollutant identification and validation in this study are largely indirect and hypothesis-generating. The initial CTD-based screening may not capture all relevant pollutants or exposure contexts, and subsequent toxicity predictions and molecular docking are computational, not replacements for direct experimental validation. While binding free energies suggest stable *in silico* interactions, they do not confirm physical binding in biological systems, nor do they account for protein dynamics, cellular microenvironment, or pollutant metabolism. Moreover, our *in vitro* PFOS experiments only demonstrate correlative upregulation of TAGLN2 mRNA and increased proliferation, without proving direct binding or mechanistic dependency. Real-world exposures involve pollutant mixtures, whereas this study examined individual compounds in isolation. Thus, these computationally derived findings should be viewed as preliminary candidates requiring further validation through direct binding assays (e.g., SPR, ITC), *in vivo* models, and mixture effect assessments.

Fifth, the interpretation of hazard ratios (HRs) and their confidence intervals (CIs) warrant further discussion. The HRs presented in Tables 1–3 represent the relative change in pulmonary arterial hypertension (PAH) risk per one standard deviation increase in genetically predicted protein abundance, gene expression, or methylation level (for CpG sites). For example, the HR of 9.00 for TAGLN2 protein indicates that a one standard deviation increase in genetically predicted TAGLN2 abundance is associated with a 9-fold increase in PAH risk; however, its wide 95% CI (1.52–53.16) reflects the modest number of PAH cases (n = 301) in the FinnGen-GWAS, leading to imprecise point estimation. Nevertheless, the lower bounds of all significant HRs are far from 1 (>1 or <1), suggesting consistent directional effects.

Sixth, future experimental validation is required to strengthen the current findings. Our study validated only one pollutant (PFOS) in a single cell type (HPASMCs). The other seven computationally predicted pollutants, dibutyl phthalate, bisphenol B, bisphenol S, bisphenol F, benzo[a]pyrene, aflatoxin B1, and nicotine, remain untested. Future studies should examine whether these compounds similarly upregulate TAGLN2 expression and promote HPASMC proliferation … Additionally, replicating the experiments in other relevant cell types (e.g., human pulmonary artery endothelial cells, primary PASMCs from different donors, or rodent PASMCs) would help assess the generalizability of our observations. Finally, animal models (e.g., chronic PFOS exposure in rodents combined with genetic or pharmacological modulation of TAGLN2) are essential to evaluate the *in vivo* relevance of the TAGLN2-proliferation axis in pulmonary vascular remodeling.

## Conclusion

In conclusion, this study integrates multi-omics data, SMR analysis, and reverse network toxicology to prioritize TAGLN2 as a genetically associated candidate in PAH. The SMR analysis revealed suggestive associations of TAGLN2 with PAH risk at the levels of DNA methylation, gene expression, and protein abundance. Increased expression and protein levels of TAGLN2 were associated with higher disease risk, whereas specific methylation sites (e.g., cg13892570, cg16107628) showed statistically inverse associations, though direct regulatory effects on TAGLN2 expression remain unproven. Eight environmental pollutants were computationally predicted as potential modulators of TAGLN2. *In vitro* experiments showed that PFOS treatment was associated with upregulation of TAGLN2 expression and increased HPASMC proliferation, but these findings are correlative and do not establish a causal mechanism. Nonetheless, due to the lack of functional perturbation experiments, these findings should be considered hypothesis-generating. This research provides a preliminary framework for exploring potential gene-environment interactions in PAH and lays a theoretical basis for future mechanistic and environmental risk studies.

## Data Availability

The original contributions presented in the study are included in the article/Supplementary Material, further inquiries can be directed to the corresponding author.
